# Attentional Bias in Humans Toward Human and Bonobo Expressions of Emotion

**DOI:** 10.1177/14747049211032816

**Published:** 2021-07-28

**Authors:** Mariska E. Kret, Evy van Berlo

**Affiliations:** 1 Leiden University, Institute of Psychology, Cognitive Psychology Unit, CoPAN lab, Wassenaarseweg, Leiden, Zuid-Holland, The Netherlands

**Keywords:** emotion perception, attentional bias, evolution, cross-species, gender, age

## Abstract

Correctly recognizing and efficiently attending to emotional situations are highly valuable skills for social species such as humans and bonobos, humans' closest living relatives. In the current study, we investigated whether humans perceive a range of emotional situations differently when these involved other humans compared to bonobos. A large group of children and adults participated in an emotion perception task and rated scenes showing either bonobos or humans in situations depicting distressed or aggressive behavior, yawning, scratching, grooming, playing, sex scenes or neutral situations. A new group of people performed a dot-probe task to assess attentional biases toward these materials. The main finding is that humans perceive emotional scenes showing people similarly as emotional scenes of bonobos, a result reflecting a shared evolutionary origin of emotional expressions. Other results show that children interpreted bonobos’ bared teeth displays as a positive signal. This signal is related to the human smile, but is frequently seen in distressed situations, as was the case in the current experiment. Children may still need to learn to use contextual cues when judging an ambiguous expression as positive or negative. Further, the sex scenes were rated very positively, especially by male participants. Even though they rated these more positively than women, their attention was captured similarly, surpassing all other emotion categories. Finally, humans’ attention was captured more by human yawns than by bonobo yawns, which may be related to the highly contagious nature of yawns, especially when shown by close others. The current research adds to earlier work showing morphological, behavioral and genetic parallels between humans and bonobos by showing that their emotional expressions have a common origin too.

Social animals, like humans and great apes, spend a considerable amount of their time among conspecifics. In interactions with them, they produce, observe and exchange emotional expressions. Because emotional expressions provide relevant information and inform subsequent actions, they are efficiently processed; they readily attract the attention of observers and are recognized easily (e.g. Ekman, 1984; Frijda et al., 1989). The underlying mechanisms of producing and recognizing emotional expressions are deeply grounded in our evolutionary past and it is therefore not surprising that important parallels in emotion processing exist between humans and other great apes (Darwin, 1872; Kret et al., 2020). The majority of studies investigating emotion recognition and attentional biases toward emotions in humans has made use of isolated facial expressions as stimulus materials. However, emotions and facial expressions of emotion alike, are embedded in complex social scenes involving multiple individuals. Moreover, the literature is troubled with inconsistencies regarding gender (Kret & de Gelder, 2012a) and age differences (Van Rooijen et al., 2017). The goal of the current study was to assess the effect of naturalistic emotional scenes on the perception of emotional expressions. To address the aforementioned inconsistencies, we opted for a large community sample consisting of males and females and children as well as adults. Crucially, we compared the processing of scenes showing humans and apes in similar emotional scenarios to address the question of whether effects are human-emotion specific, or more generally linked to emotions and similar when observing emotions expressed by apes. Congruent findings across human and ape scenes would further support their shared evolutionary basis.

There is discussion in the human emotion literature about whether or not emotions and their expressions can be put into categories. Research showing that humans across the world can assign emotion labels to prototypical facial expressions suggests that such distinct categories exist (Ekman, 1984). However, contrasting literature shows that the same emotional expression can be interpreted differently based on context (Kret & de Gelder, 2010, 2012a, 2013; Kret & Straffon, 2018). Crucially, emotional expressions are perceived differently when posed by actors as compared to when real-life stimuli are employed (e.g., Motley & Camden, 1988; McLellan et al., 2010). Despite this evidence, the majority of studies has been using posed expressions, ignoring the fact that these prototypical expressions are not commonly observed in real life. For example, how often do we see the facial expression of fear? The smile, an expression that is common in real life, often gets the simplified label “happiness” in emotion research. However, the smile is a communicative signal with meanings ranging from greeting another person, an expression of love, to a contemptuous smile or a sign of nervousness (Kret, 2015; Kret et al., 2020). Given that emotions can be expressed in multiple ways, the use of isolated stimuli in research leads to perceptual confounds, and therefore muddies the interpretation of results. Take for instance the observed attentional bias toward smiling faces. The question is whether we can be sure this attentional bias is related to the smile or whether it can be attributed to low-level characteristics of the included stimulus materials such as the exposure of the white teeth with the smile (Blanco et al., 2017). Further discussion about these debated topics is beyond the scope of this article. What we aim to do here is to take a step aside and study how emotional expressions are perceived from a large number of complex, naturalistic scenes depicting real life, authentic emotional situations, where emotional expressions are embedded within rich contextual features, thereby partly circumventing the above problems. This approach is ecologically more valid since in the natural world emotional expressions are always embedded in a scene (Kret & de Gelder, 2010).

The experience and expression of emotions is heavily reliant on contextual cues (Hess et al., 2016). The interpretation of facial expressions relies on contextual information, such as body language (Kret & de Gelder, 2013; Kret, Roelofs, Stekelenburg, & de Gelder, 2013; Kret, Stekelenburg, Roelofs & de Gelder, 2013), outgroup cues (Kret & de Gelder, 2012b; Kret & Fischer, 2018; Liedtke et al., 2018) and the global processing of a social scene (Ngo & Isaacowitz, 2015; Righart & de Gelder, 2008a; Van den Stock et al., 2013). Likewise, the perception of emotional body language is influenced by the facial expression (Kret & de Gelder, 2012a) and the social context (Kret & de Gelder, 2010). In a study where participants were asked to explicitly label a person’s emotional expression, contextual influences on emotion perception varied based on the type of contextual cue, cue relevance, and the perceiver’s age (Ngo & Isaacowitz, 2015). EEG studies have demonstrated that the integration of these different pieces of information occurs early in the processing stream, further underscoring its relevance (de Gelder et al., 2006; Righart & de Gelder, 2005, 2008b). Moreover, the perceived valence and arousal from such scenes is modulated by gender (for a review, see Kret & de Gelder, 2012b) and age (e.g. Backs et al., 2005). The dot-probe paradigm is widely used to measure attentional biases toward certain stimulus categories such as expressions of emotion. An advantage is its implicitness and simplicity, so that it can be administered in young children or nonhuman primates (Kret et al., 2016). A recent meta-analysis (of 38 articles including 4,221 children) confirmed that children show a significantly greater bias to threat-related stimuli than to neutral stimuli and this effect was stronger in children with high anxiety, a difference that further increased with age (Dudeney et al., 2015).

In typical emotion perception tasks it is impossible to pull apart effects of emotion as an isolated construct from effects of emotions expressed by a specific species. The latter is commonly assumed, but whether effects are specific to the emotional expressions of other humans can only be investigated by directly comparing biases toward emotional expressions of other species. On the one hand, one could argue that it is easier to embody the emotional expressions from conspecifics than from other species with very different bodies. On the other hand, as Darwin (1872) already noted, the similarities in the expression of emotion across species are high and may render the former argument trivial. An earlier preliminary study gives some insight into this question (Kret et al., 2018). In the study, participants performed a dot-probe task with threatening or neutral expressions expressed only by adult male humans and chimpanzees. The results showed a significant bias toward threat, but no interaction between threat and species; suggesting that processing emotional expressions may not rely on the species of the expressor. This finding further supports the evolutionary argument. An alternative explanation is that attentional capture from threat is also functional: humans benefit from quickly detecting and responding to threatening stimuli irrespective of whether it is displayed from a human or chimpanzee. Nevertheless, whether the same principle holds for positive expressions remains uncertain. Positive emotions, child or female models were not included so the generalizability of the findings is uncertain. For instance, humans would likely perceive expressions of sexual arousal as more relevant or interesting when expressed by a human compared to a chimpanzee. In contrast, the image of two playing apes expressing joy might attract human attention. Indeed, it is common in zoos for apes to end up tangled in a play of tag or peek-a-boo with human children. In the current study, we aim to disentangle the effect of sender species on emotional attentional biases. To that extent, the bonobo provides an excellent model. Not only is it our closest living relative (together with the chimpanzee we shared a common ancestor that lived roughly 6 million years ago) and shows very similar musculature of the face and body (Diogo, 2018), it also is a very rare species that people do not get to see often. There are only two zoos in the Netherlands that house a group of bonobos, so except for frequent visitors of these zoos, people in general have had few or no learning experiences with these animals.

An important factor that might account for discrepancies in attentional biases toward emotions is individual differences, such as age and gender. As humans age, certain situations might become more prevalent or important. For instance, older and more experienced individuals might quickly see opportunities that children do not, like recognizing that someone is flirting. Similarly, children might detect other opportunities that adults fail to detect, such as recognizing playful intentions in potential play mates. Gender also influences the ability to detect emotional expressions. Specifically, biases toward threat tend to be larger in males than females, especially when they experienced violent environments (e.g., Kret & de Gelder, 2013; Shechner et al., 2012). In our earlier described study, gender or age differences did not modulate the observed attentional bias toward emotions (Kret et al., 2018). However, that study only included greyscale fearful and aggressive expressions of males and therefore this question needs to be investigated in an experiment including multiple emotions expressed by both genders. Whether attention is differentially captured depending on age or gender is part of what will be investigated in the current study.

The current study investigates how people perceive emotions from a large number of naturalistic scenes showing humans or bonobos. With rating scales, we gain insight into how participants perceive the observed images in terms of valence and arousal. Using dot-probe tasks, we address the question of which types of emotion scene capture attention most. Furthermore, using a large community sample allows us to unveil possible effects of age and gender.

## Method

### Participants

Participants consisted of a large group of visitors and employees of a Dutch zoo (Apenheul Primate Park, Apeldoorn, the Netherlands). The sample size was the result of a fixed number of days of testing agreed with the zoo. One part of the participants participated in a task assessing the perceived emotional valence and arousal of a series of stimuli, the other part in a dot-probe experiment. There were two reasons for deciding à priori to create separate groups for adults and children. First, children and adults took part in slightly different versions of the task: children did get trials with pictures showing bullying behavior, but no overt aggressive scenes and no sex scenes. Second, based on our experience with testing visitors in the zoo, we knew in advance that there would be relatively few 14–18 years old as most families visit with younger children and that age would not be normally distributed. Table 1 summarizes all demographic information. After indicating interest in participating in one of the experiments, participants gave informed consent. The study was approved by the Ethics Committee of Leiden University (CEP17-0213/74 for adults, CEP17-0604/222 for children). All participants were debriefed after the completion of the study.

**Table 1.** Demographic Information of the Participants.

			Gender		Age			
Type of task	Species on stimuli	Participants	Female	Male	Mean	SD	Min	Max
Emotion Perception	Human	Adult	53	47	36.28	15.61	18	75
		Child	13	13	8.41	2.00	5	12
	Bonobo	Bonobo keeper	4	1	37.88	9.62	26	50
		Adult	201	157	30.50	13.54	18	84
		Child	48	53	9.93	3.05	4	17
Dot-probe	Human	Adult	84	68	33.94	13.08	18	74
		Child	69	80	9.360	2.67	3	17
	Bonobo	Adult	153	135	37.45	16.13	18	78
		Child	51	69	9.28	2.90	4	17

### Experimental Stimuli

The stimulus materials have been used in two previous studies with bonobo subjects. In the first study, Kret et al. (2016) showed bonobos emotional pictures including sex, grooming, yawning, panthoot, interactions with food, play, and distress (Figure 1 in Supplements). The authors observed that bonobos’ attention was immediately captured by emotional scenes. In a subsequent study, van Berlo, Bionda, and Kret (2021) used all new, but similar stimulus materials. In their study, the scenes showing food and panthoots were dropped as these showed hardly any effect in the first study. In addition, based on behavioral observations that were made during the first study, they decided to include scenes where individuals were scratching, which occurs under arousal (Maestripieri et al., 1992). The findings of van Berlo replicated the effect observed in Kret et al. (2016), suggesting that bonobos recognize the emotional expressions of conspecifics and that these are thus readable and distinctive from the neutral scenes.

**Figure 1.**
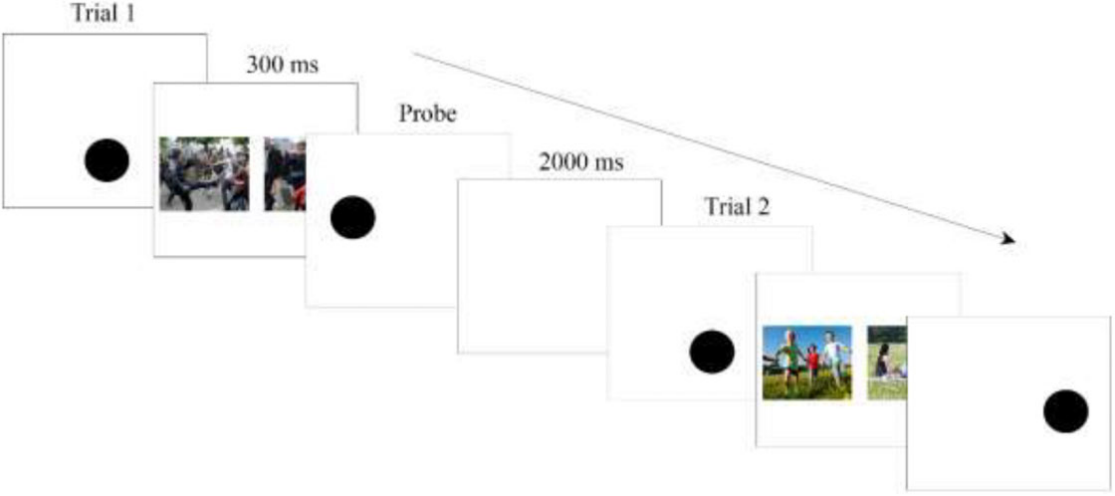
Trial outline of the dot-probe.

In the current study, we investigated how humans perceive scenes which are relevant for bonobos and thus kept these scene categories consistent with our previous work. To examine the differences in attentional bias when expressions are produced by bonobos and humans, we created a human dataset equivalent to the bonobo one (van Berlo et al., 2020) (Figure 2 in Supplements). The stimulus set included people yawning or scratching, two highly contagious behaviors (Campbell & de Waal, 2011; Palagi et al., 2014; Swithenbank et al., 2016). Play scenes showed adults and/or children immersed in playful interactions. For the category grooming we used images of humans embracing each other or combing or braiding each other’s hair. The category sex showed a man and a woman in an intense, erotic embrace (without depicting their genitalia). The neutral category showed people walking or sitting. The distress category depicted crying individuals. The category aggression differed for adults and children; adults viewed violent scenes (e.g., fist-fights) and children saw images of bullying scenes. Adults and children further viewed the same stimulus set, excluding the category sex for children. In the bonobo version, all scenes showing distress showed the bared teeth display, a facial expression that is frequently used to signal submission and shows commonalities with the human fearful facial expression and to the expression of the smile (Kret & Straffon, 2018). The distressing images had a negative valence, but did not include overt aggression. Omission of overt aggression was due to the fact that bonobos are a relatively non-aggressive species, therefore there was no sufficient amount of aggressive images available. All stimuli were sized 400 × 330 pixels.

### Procedure

The task was displayed on a Dell S2240 Tb touchscreen monitor (21.5 inch, 1920 × 1080 resolution, 60 Hz refresh rate) and ran on a Dell OPTIPLEX 990 desktop computer using E-prime 2.0. The experimental set-up was placed in a quiet, public corner of the indoor bonobo enclosure and facing the wall to minimize distractions (see Figure 3 in Supplements).

### Emotion Perception Task

Adult participants were asked to rate the valence and arousal of the human (n = 126) and bonobo (n = 467) pictures on 1–7 rating scales (1 being negative or low arousing; 7 being positive or high arousing). Children used the Self-assessment manikin (line drawing of a cartoonish human figure with a facial expression ranging from very negative to very positive Lang, 1980).

### Emotional Dot-Probe Task

Based on the scores of the emotion perception task, we selected 10 scenes per emotional category that were a) best recognized regarding valence and b) most emotionally arousing. The selected pictures were used as stimuli in the human-scene dot-probe task. For the bonobo-scene dot-probe task, stimuli were selected using the validation data of experts gathered during previous studies with bonobos (Kret et al., 2016; Van Berlo et al., 2020).

During the dot-probe task, a trial started with the presentation of a black dot centered horizontally in the lower quadrant of the display that remained visible until response (i.e., tapped) (see Figure 1). Following a response, two images (emotional vs. neutral) were simultaneously presented on the left and right quadrants of the display (50% chance) for 300 ms and replaced by a black dot either on the left or right location of the display (50% chance). Every trial ended with an inter-trial interval (ITI) of 2,000 ms. The location of the dot and stimuli were balanced. Trials were presented in a pseudo-randomized manner. There were two different versions of the bonobo scene dot-probe task. Version 1 included 45 trials and Version 2 (which had one additional emotional category, i.e. “scratching”) included 60 trials. The human scene dot-probe task included two threat categories, namely distress and aggression. For children there was an equivalent category of pictures for aggression showing bullying behavior.

### Statistical Analyses

All data were analyzed using generalized mixed modeling with experimental trials nested within subjects (SPSS; version 25). A random intercept of participant was included in all analyses. Significant interactions and main effects of Emotion Category were followed-up using *t-*tests. In order to reduce type I errors, the *p*-value was set to 0.01. Follow-up simple contrasts are Bonferroni-corrected. Only the significant effects of major theoretical interest are visualized. We start with reporting main effects, followed by two-way interactions, followed by higher order interactions, in the order of significance (most significant effects first).

### Rating Scales

The dependent variables in this emotion perception task were Emotional Valence and Intensity (1–7 scaled ratings). We used a linear distribution function to model the data, as they were normally distributed. Three separate models were conducted with minor differences. All models included the fixed factors of Age Group (Child/Adult), Gender (Male/Female), Emotion Category (Sex [adults only]/Groom/Play/Scratch/Distress/Aggression [adults and human scenes only]/Yawn/Neutral) and Species Scene (Human/Bonobo). In the first model, we examined the effect of age, gender, expressor species, and emotional display on intensity ratings. Therefore, we omitted the categories Sex and Aggression since a) children did not see these images and b) the aggression category was not included in the bonobo scenes. Therefore, this model included scenes depicting grooming, yawns, play, distress, and scratching. In the second model, we zoomed in on human adults and therefore added the emotion category Sex. In the third model, we included only human adults and human scenes, and added the emotion category Aggression.

### Attentional Bias

The reaction times were filtered with the following procedure. First, age categories were created using 5-year bins (e.g., 0–5 years old; 5–10 years old) and trials with extremely fast (<250 ms) or extremely slow responses (>5,000 ms) were excluded. Next, the trials exceeding 2.5 mean absolute deviations (MAD) from the mean per age bin were excluded. The reason for this procedure is that very young children and older people tend to be a lot slower on this task than those with an average age. After applying these criteria, 10.14% of the data was excluded. The data were modeled using a Gamma distribution (loglink function), given the typical skewness in RT data.

The statistical procedure was similar to the analysis of the rating scales, unless stated otherwise. In the model for the dot-probe task, two additional factors were included, a) Congruency (probe position replacing the emotional stimulus or not) and b) Distance of dominant hand to the screen (Short/Long). This latter factor is added as a control to account for the shorter distance for a left-handed person from the left hand to the left side of the screen and for a right-handed person using the right hand to the right of the screen compared to left to right and vise-versa.

## Results

### Perceived Valence of Human and Bonobo Scenes

In a first analysis where the emotion categories Sex and Aggression were excluded, participants (adults and children), showed a main effect of Emotion Category (*F*(1, 11.36) = 124.86, *p* < .001). Play was most positively rated out of all categories, followed by groom, neutral, yawn, distress and scratch (the latter being least positively rated of all). All emotion categories differed from the neutral scenes in the expected direction (*p*s ≤ .001). A main effect of Species showed that in general, bonobo scenes were perceived more positively than human scenes (*F*(1, 11.36) = 11.83, *p* = .001).

An interaction between Emotion Category and Species (*F*(5, 11.36) = 87.76, *p* < .001) showed that human scenes received more extreme ratings than bonobo scenes. That is, positive emotions received more positive ratings and negative ones more negative ratings when expressed by humans rather than bonobos. This was significant for the categories Play (*t*(12.54) = 6.37, *p* < .001) and Distress (*t*(11.36) = 20.63, *p* < .001), with a trend toward significance in the same direction for scratching (*p* = .031). In contrast, another trend was observed for yawning behavior, which was rated somewhat more negatively in the bonobo compared to human scenes, possibly because of the visibility of the canines (*p* = .024). There were no differences for neutral (*p* = .922) or grooming scenes (*p* = .551).

Further, an interaction between Emotion Category and Age (*F*(5, 11.36) = 7.81, *p* < .001) showed that compared to adults, children differentiated the categories less based on valence. Specifically, they gave less negative evaluations of the negative scenes (this was significant for the category Distress *t*(11.36) = 3.64, *p* < .001, with trends in the same direction for scratch (*p* = .034) and yawn (*p* = .014)) and less positive ratings following positive scenes (which was significant for grooming *t*(11.36) = 3.44, *p* = .001 and showed a trend in play (*p* = .023).

**Figure 2.**
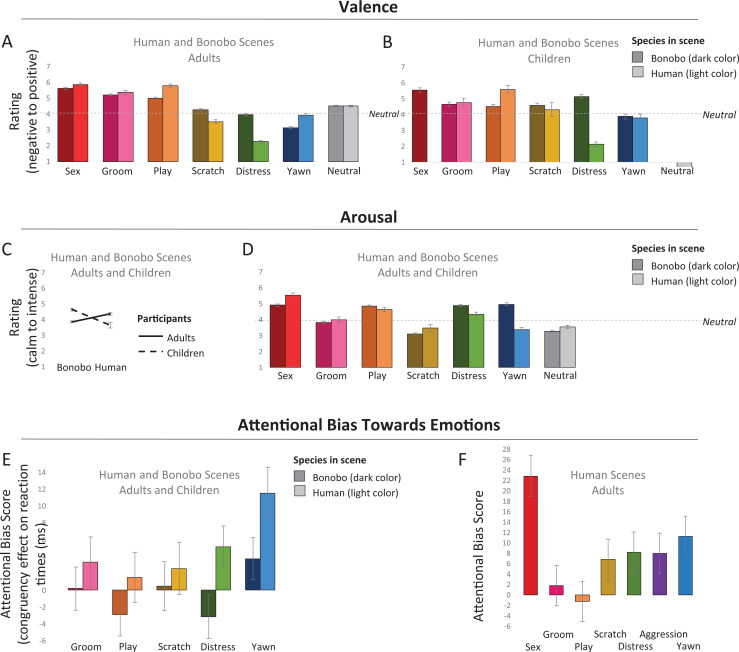
Strongest effects of valence (A: adults; B: children) and arousal ratings (C, D) and attentional bias (E, F: the neutral category is at zero).

A three-way interaction between Age, Species, and Emotion Category (*F*(5, 11.36) = 10.86, *p* < .001) demonstrated that this effect was driven by the bonobo scenes. There were no differences between adults and children in the perception of valence from human scenes (*p*s ≥ .023 [for distress]). In contrast, the bonobo scenes were perceived differently. Specifically, compared to adults, children perceived bonobo scenes showing grooming and play less positively (grooming: *t*(11.36) = 4.26, *p* < .001; play: *t*(11.36) = 3.20, *p* = .001) and scenes depicting individuals yawning less negatively (yawning: *t*(11.36) = 5.15, *p* < .001). Further, adults gave all positive emotional scenes more positive ratings than the neutral scenes and all negative scenes more negative ratings than the neutral ones. Children, in contrast, did often not differentiate the emotional scenes from the neutral ones in terms of valence. However, they did rate human yawn (*p* = .002) and distress scenes (*p* < .001) more negatively, and play (*p* < .001) more positively than neutral scenes. Regarding the bonobo scenes, they rated scenes depicting yawning bonobos more negatively than neutral (*p* < .001) and distress scenes more positively than neutral scenes (*p* < .001). See Figure 2.

In a second analysis we focused on human adults, adding the category Sex. Within the adult sample, the main effect of Emotion Category remained significant (*F*(1, 10.54) = 357.92, *p* < .001) with the ordering of the categories being similar as before, except that the category Sex received the most positive rating. Within human adults and with the category Sex included, an effect of Gender emerged, with male participants giving more positive ratings than females (*F*(1, 10.54) = 13.58, *p* < .001). The earlier observed effect of Species disappeared (*p* = .239), showing that adults perceive human and bonobo scenes to be equal in valence overall. The interaction between Emotion Category and Species was maintained (*F*(6, 10.54) = 64.88, *p* < .001), and the interpretation was not altered. Interestingly, an interaction between Emotion Category and Gender (*F*(7, 10.54) = 6.73, *p* < .001) showed a significant gender difference in several emotion categories, most prominently in the category Sex (*t*(10.54) = 4.39, *p* < .001), which men rated with almost half a point more positively than women. Further, distress and yawn images were perceived more negatively by women compared to men (distress: *t*(10.54) = 3.32, *p* < .001; yawn: *t*(10.54) = 2.75, *p* = .006).

In a third analysis within human adults, we zoomed in on the human scenes specifically, and added the category Aggression. The results showed a main effect of Emotion Category (*F*(1, 2.62) = 462.22, *p* < .001) and an interaction between Emotion Category and Sex (*F*(1, 2.62) = 3.587, *p* = .001). Of all categories, Aggression was perceived most negatively and Sex most positively. Further, the only gender difference that remained significant after having excluded the bonobo scenes from the analysis was the category Sex, which males rated as more positively than females (*t*(2.62) = 2.85, *p* = .004).

### Perceived Arousal of Human and Bonobo Emotional Scenes

In a first analysis where the emotion categories Sex and Aggression were excluded, participants (adults and children), showed a main effect of Emotion Category (*F*(1, 11.03) = 111.39, *p* < .001). Ordering the categories from high to low based on the arousal ratings led to the following order: Play (most arousing out of all categories), Distress, Yawn, Groom, Neutral, Scratch (rated as least arousing of all). Apart from the category Scratch (*p* = .110), all emotion categories differed significantly from neutral (*p*s < .001).

A gender difference showed that women gave higher intensity ratings than men (*F*(5, 11.03) = 29.11, *p* < .001).

An interaction between Emotion Category and Species (*F*(5, 11.03) = 31.39, *p* < .001) showed that human observers judged specific emotion categories differently when the scenes depicted bonobos compared to humans. Most strikingly, while yawning was considered the least arousing emotion category of the human scenes, it was the most arousing one of the bonobo scenes. For the rest of the expressions, the overall pattern was similar although the bonobo scenes received higher arousal ratings than human scenes for the categories Yawning (*t*(11.03) = 8.71, *p* < .001) and Distress (*t*(11.03) = 4.03, *p* < .001), but lower arousal ratings than human scenes for the category Neutral (*t*(11.03) = 6.84, *p* < .001 [with a similar trend for scratching (*p* = .05)]).

Further, an interaction between Species and Age Group (*F*(1, 11.03) = 110.02, *p* < .001) showed that while children rated bonobo scenes as more intense than human scenes (*t*(11.03) = 6.14, *p* < .001), the opposite was true for adults, who rated human scenes as more intense (*t*(11.03) = 10.90, *p* < .001). On similar lines, children rated bonobo scenes as more intense compared to adults (*t*(11.03) = 14.72, *p* < .001) while the opposite was true regarding the human scenes (*t*(11.03) = 4.59, *p* < .001).

An interaction between Age Group and Emotion Category (*F*(5, 11.03) = 12.45, *p* < .001) showed that compared to adults, children gave higher intensity ratings to scenes depicting play (*t*(11.03) = 4.00, *p* < .001) or neutral actions (*t*(11.03) = 9.37, *p* < .001), but lower arousal ratings to distress scenes (*t*(11.03) = 2.61, *p* = .009).

The two-way interactions were further qualified by two three-way interactions. First, there was an interaction between Age Group, Species and Emotion Category (*F*(5, 11.03) = 21.87, *p* < .001). Follow-up tests revealed that compared to adults, children gave higher ratings following most bonobo scenes (all categories *p*s < .001, except scratching; *p =* .18, although numerically in the same direction). Zooming in on the human scenes showed that compared to adults, children perceived the distress scenes as less intense (*t*(11.03) = 8.89, *p* < .001) and the neutral scenes as more intense (*t*(11.03) = 4.74, *p* < .001). Further, while adults perceived all but the yawn scenes as more intense when showing humans compared to bonobos (*p*s < .001, but with an opposite effect for yawning [*p* < .001]), children perceived play (*t*(11.03) = 3.76, *p* < .001), distress (*t*(11.03) = 8.24, *p* < .001) and yawning (*t*(11.03) = 7.68, *p* < .001) more intensely from scenes showing bonobos rather than humans. A final set of comparisons showed that almost all emotion categories were perceived as more intense than neutral (*p*s < .003) *except* scratching bonobos (perceived by adults *p* = .77 with an opposite effect in children *p* = .003), and in children, scratching humans (*p* = .085) or grooming humans (*p* = .933).

Finally, there was a three-way interaction between Sex, Emotion Category and Species (*F*(5, 11.03) = 5.69, *p* < .001). Follow-up tests revealed that compared to men, women gave higher arousal ratings for the following bonobo scenes: grooming (*t*(11.03) = 6.47, *p* < .001), yawning (*t*(11.03) = 4.09, *p* < .001), play (*t*(11.03) = 3.01, *p* = .003), neutral (*t*(11.03) = 3.75, *p* < .001) and the following human scenes: distress (*t*(11.03) = 4.57, *p* = .002), yawning (t(11.03) = 2.92, *p* = .004 and neutral *t*(11.03) = 2.91, *p* < .001). Also, while both males and females gave higher intensity ratings following yawning bonobos versus humans (males *t*(11.03) = 7.14, *p* < .001; females *t*(11.03) = 6.57, *p* < .001) and lower ratings following neutral bonobos versus humans (males: t(11.03) = 4.72, *p* < .001; females: *t*(11.031) = 6.10, *p* < .001), males also gave higher ratings following distressed bonobos versus humans (*t*(11.03) = 4.81, *p* < .001) and lower ratings following grooming *t*(11.03) = 3.79, *p* < .001 and playing *t*(11.03) = 2.33, *p* < .001) bonobos versus humans. Although both males and females generally gave higher arousal ratings to the emotional scenes compared to the neutral ones, this pattern was a bit stronger in men. In men, this was significant for all categories (*p*s ≤ .001) except scratch (bonobo scenes: *p* = .045; human scenes *p* = .466) and human yawns (*p* = .082). In women, apart from generally perceived higher intensity from the emotional compared to neutral scenes (*p*s ≤ .001), no differences were found in the category scratch (bonobo scenes: *p* = .036; human scenes *p* = .490), and of the human scenes the categories grooming (*p* = .225) and yawn (*p* = .850).

In a second analysis, we focused on human adults, adding the category Sex, since children did not have this category. Within the adult sample, the main effect of Emotion Category remained significant (*F*(1, 10.13) = 289.23, *p* < .001) with the ordering of the categories almost being identical as before, except that the category Sex received the most positive rating of all. The earlier observed main effect of Species was maintained, showing higher ratings for the human scenes (*F*(1, 9.52) = 289.23, *p* < .001). Similarly, the gender difference was maintained as well (*F*(1, 10.13) = 22.71, *p* < .001).

The interaction between Emotion Category and Species was also maintained (*F*(6, 10.13) = 28.50, *p* < .001), and the interpretation was the same.

An interaction between Emotion Category, Species and Sex (*F*(7, 10.13) = 4.25, *p* < .001) showed a significant gender difference in several emotion categories. Specifically, human distress, human and bonobo yawn images as well as neutral, grooming and playful bonobo scenes were perceived more intensely by women compared to men (human distress: *t*(10.13) = 3.74, *p* < .001; human yawn: *t*(10.13) = 2.84, *p* < .001; bonobo yawn: *t*(10.13) = 3.75, *p* < .001; neutral bonobo scenes: *t*(10.13) = 4.52, *p* < .001; bonobo grooming *t*(10.13) = 6.75, *p* < .001) and bonobo play *t*(10.13) = 3.71, *p* = .002).

In a third analysis in human adults, we zoomed in on the human scenes specifically, and added the category Aggression. The results showed a main effect of Emotion Category (*F*(1, 2.62) = 141.24, *p* < .001) and an interaction between Emotion Category and Sex (*F*(1, 2.62) = 2.22, *p* = .008). Of all categories, Aggression was perceived most intensely and Neutral least. Further, the only gender difference that remained significant after having excluded the bonobo scenes from the analysis was the category Distress, with females rating these scenes as more intense than males (*t*(2.62) = 2.78, *p* = .006).

### Attentional Bias Toward Human and Bonobo Emotional Scenes

Reaction times were analyzed in a generalized mixed model with the fixed factors Age Group, Gender, Species Scene, Emotion Category, Congruency (i.e. the probe appearing behind the emotional scene or not), their interactions, and in addition Dominant Hand Distance (i.e. location of probe in relation to handedness of participant). Again by using a backward elimination method, we came to the final, most parsimonious and best-fitting model that included a subset of these factors. For brevity, only significant effects that include the factor Congruency are described in the text.

In a first analysis where the categories Sex and Aggression were excluded, participants (adults and children), showed a main effect of Congruency (*F*(1, 29.07) = 6.78, *p* = .009), demonstrating that reaction times were faster when the probe replaced an emotional compared to a neutral scene, indicating heightened attention for the emotional category. An interaction between Congruency and Species (*F*(1, 29.07) = 6.84, *p* = .002), showed that the Congruency effect was significant for human scenes (*t*(29.07) = 3.76, *p* < .001), but not for bonobo scenes (*p* = .698, see Figure 2E). There were no gender differences observed (*p*s = .441 in a pre-final model) and Age Group did not significantly modulate the congruency effect either (*p* = .053, trending toward a larger effect in adults).

In a second analysis, we focused on human adults, adding the category Sex. After adding this factor, the main effect of Congruency was maintained (*F*(1, 15.45) = 8.17, *p* = .004). In addition, without the child participants and with the extra category of Sex, the previously significant interaction between Species and Congruency was rendered insignificant (*p* = .051), which replicates our earlier findings, showing that adults attend to emotions quickly, regardless of the species that expresses them (Kret et al., 2018).

Third, we zoomed in on human adults further, and specifically on their attentional biases toward human scenes. Here we had an additional emotion category, being Aggression. This analysis showed a main effect of Congruency (*F*(1, 5.71) = 13.02, *p* < .001) and an interaction between Congruency and Emotion Category (*F*(1, 6.65) = 3.80, *p* = .001). Significant congruency effects were observed in the categories Sex (*t*(6.65) = 5.75, *p* < .001) and Yawn (*t*(6.65) = 2.89, *p* = .004), trends toward significance were observed for Distress (*p* = .037), Aggression (*p* = .041) and Scratch (*p* = .084) and no effects for Grooming (*p* = .641) and Play (*p* = .742, see Figure 2F).

### Discussion

Emotional expressions are pivotal to our social life. Correctly recognizing expressions and quickly attending to them can have life-saving consequences and long-lasting effects on social relationships. Like humans, bonobos are social species and have a rich repertoire of expressions. The goal of the current study was to investigate whether human participants perceive emotional scenes showing people similarly or differently as matched emotional scenes of bonobos. Specifically, how do laypeople perceive human scenes compared to scenes depicting bonobos? Overall, the results show more similarities than differences between the perception of human compared to bonobo scenes, especially in adult observers. In general, participants were able to assign appropriate valence and arousal ratings to the emotional scenes and also showed an attentional bias toward them. Interestingly, they did not only do so for the human scenes, but also for the bonobo scenes. In addition to these overall findings, the perception of the scenes differed between adults and children and females compared to males, which can potentially be attributed to different levels of experience with certain expressions or to differences in which emotional expressions are most relevant for specific individuals. In the following section, we first discuss the results regarding the valence ratings, followed by the arousal ratings and last but not least, attentional biases, reflected in the results of the dot-probe tasks.

Overall, participants’ valence ratings supported our hypothesis: positive scenes were given positive ratings and negative scenes negative ratings. Despite a similar pattern in valence ratings between human and bonobo scenes (See Figure 2A), this effect was amplified for the human scenes, particularly in child observers. This pattern demonstrates that expressions from conspecifics might indeed be easier to interpret than expressions from another species (see also Fugate et al., 2010; Kret et al., 2018). Interestingly, despite similarities in ratings between adults and children, we observed differences in the magnitude of this effect. Specifically, compared to adults’ ratings, the valence pattern was less pronounced in children. This finding is in accordance with earlier literature, showing that children’s understanding of emotional expressions is not fully developed yet (Widen, 2013). Interestingly, we found that children perceived the photographs showing bonobos in distress positively rather than negatively. The bared teeth display that was shown in scenes of the distress category is related to the smile; the latter is a ritualized version of the former (Van Hooff, 1972). Importantly, the meaning of the bared teeth display varies, and should be interpreted within the context in which it occurs. This expression can signal fear, subordinance or affiliation and the same is true in humans; a smile is not always a positive signal and can express nervousness or even contempt (Kret & Straffon, 2018). However, taking into account the context when it comes to interpreting these expressions may be something that most adults have learned but that children have not yet learned to incorporate. This interpretation remains speculative as in the current study, the human positive scenes showed smiles and laughter and these expressions never occurred in the negatively valenced scenes. Another possibly is that children’s understanding of specific emotions begins not with static images and the expressions visible in them, but with the antecedents and behavioral consequences of the emotional situation.

Of all categories, scratch was perceived most negatively and play most positively. Within the adult sample we also included sex scenes and scenes showing aggression, which received extremely positive and negative ratings respectively. Male participants in particular evaluated the human sex scenes more positively than women. Previous literature has shown that gender differences in the pleasant dimension occur only for erotica, with more positive ratings for men than women (Bradley et al., 2001). Surprisingly, these differences were very minor in the current sample. For instance, in the study by Bradley et al. (2001), women rated images of “erotic couples” or “opposite sex erotica” roughly 65% positive and men 85%. However, in our study, the percentages were about 81% for women and 87% for men. A possible explanation for this effect is that we included more women-friendly images (i.e., pictures of couples embracing, with no close-ups of their genitalia or explicit depiction of sexual acts). Indeed, previous research has shown that women report increased negative affect after viewing mainstream (i.e., male-centered) sexual material (Koukounas & McCabe, 1997) but increased positive affect after viewing women-friendly material (Laan et al., 1994; Mosher & Maclan, 1994; see also Heiman, 1977). The contextual cues provided in women-friendly sexual material facilitate their positive appraisal and might promote the detection of sexual feelings in women (Laan & Everaerd, 1995; see also Laan & Janssen, 2007). Given these findings, the observed gender differences in self-report of positive affect might be due to stimulus selection, even though men consistently tend to give higher ratings to sexual material (Janssen et al., 2003). Alternatively, social desirability factors were at play, as women might be more inclined to modulate self-reports of positive affect in response to sexual material (Morokoff, 1985; but see Brody et al., 2003). In contrast, men might overestimate their responses (Catania et al., 1990). The difference between the current and the previous study might reflect a cultural difference between the USA versus the Netherlands. For example, while naked breasts or explicit sex scenes are common in typical European movies, such scenes are less common in American movies. Although we cannot with certainty say what the different finding in the literature regarding the perception of sexual images in males as compared to females causes, a crucial consideration is that our sample consisted of people from the general public. This implies that our findings are more representative for the general population than previous laboratory-conducted sex research that typically included students.

Compared to male participants, females interpreted the human distress and yawn scenes relatively negatively. These findings are in line with earlier literature. The majority of previous work used images from the International Affective Picture System (IAPS; Lang et al., 2005), a collection of standardized and digitized color photographs that depict objects and scenes. Although considerable agreement has been found between men and women in their categorical labeling of these images to different emotions (Mikels et al., 2005), women typically assign unpleasant pictures a more negative valence rating than do men (Bradley et al., 2001; Gard & Kring, 2007).

Apart from valence ratings, we also asked participants how they perceived the scenes in terms of eliciting arousal. In general, they evaluated the emotional scenes as more arousing than the neutral scenes. Play scenes received the highest intensity scores and the scratch category the least. Interesting differences were observed in how people evaluated human compared to bonobo scenes. Specifically, while yawning was considered the least arousing emotion category of the human scenes, it was the most arousing one for the bonobo scenes. Possibly, the display of the relatively large canines of bonobos played a role here. When humans yawn, they typically do not show their teeth and the fact that they often cover their mouth with their hands, shows that it is not a socially well-accepted expression. The canines provide a likely interpretation given that a similar finding was observed for the distress category where the canines are visible as well. At the same time, such differences make it problematic for the interpretation of the current findings. Is the effect indeed due to the canines? Or is it that the opening of the mouth of a bonobo raises a greater risk of being bitten because it is more difficult to predict subsequent actions of another species that has a completely different body and that one has hardly any experience with? One species might be better at expressing a certain emotion thanks to certain physical characteristics. For example, bonobos have more bodily hair than humans and during dominance displays, these hairs my bristle, called pilorection, which makes them appear larger and perhaps get the message across better. Whether or to what extent physical differences in the face and body of different species translate into perceptual differences of their expressions is a research topic that has remained unexplored. This needs to be confirmed in a comparative study with bonobos and humans. Importantly, differences in facial musculature between bonobos and humans are negligible .

The literature is torn on the intensity between differentially valenced stimuli. Which one would be more intense: sex or aggression? Interestingly, aggressive and sexual images were, on average, perceived as most intense in our study. In contrast to earlier literature showing that men perceive sexual images as more intense compared to women (Bradley et al., 2001), we observed no such difference. However, in Bradley et al. (2001), the greatest difference was found in the valence ratings and the intensity ratings of the sex scenes differed only mildly. In the current study, a gender difference did occur in the human distress category. Females indicated to perceive these scenes as more arousing than males, which is also in line with earlier work (Bradley et al., 2001). Females also perceived some of the bonobo scenes as more arousing then men, including scenes showing yawns, grooming and play. However, a gender difference was also observed in the neutral bonobo scenes so this might also just reflect a more general gender difference in self report. Indeed, in general, women gave higher intensity ratings than men, as demonstrated by a main effect (see also Bradley et al., 2001; for a review, see Kret & de Gelder, 2012b).

The analysis of the arousal ratings also yielded some interesting results when comparing adults and children. Intriguingly, while adults perceived the human scenes as more intense than the bonobo scenes, an opposite effect was observed in children. To what extent the zoo setting influenced these results is a factor that may be taken into consideration in a future study. However, the effects were not general, which speaks against such an explanation. More specifically, children gave higher intensity ratings to bonobo play scenes. Moreover, compared to children, adults gave higher intensity ratings for scenes depicting human distress, but lower ratings for bonobos in distress. As we will see in the following section, none of these effects were linked to specific age-related attentional biases.

The results of the dot-probe task show that in line with our earlier study, humans show a robust attentional bias toward emotions (Kret et al., 2018). The attentional bias was stronger when humans observed emotions that were expressed by other humans compared to expressions by bonobos. Interestingly, while humans’ attention was immediately captured by images of yawns, this was not driven by the visibility of the larger canines of bonobos which could potentially pose a high threat. Instead, attention capture was most pronounced when seeing other humans yawn. Yawns are extremely contagious and yawn contagion seems to work particularly well between close others, possibly due to heightened attention (Massen & Gallup, 2017). At first sight, this increased attentional bias toward human emotions seems to be in contrast to the findings of our earlier study (Kret et al., 2018). However, the current study deviates from our previous work in several important ways. Most crucially, the included stimulus materials consist of naturalistic scenes instead of isolated and greyscale neutral or threatening body expressions of male apes and humans in our earlier study. More importantly, within the adult sample of our study, the interaction between species and congruency disappeared. This suggests that the perception of other species emotional expressions is not fully developed yet in children, which is also in line with their dampened valence effects. As a following, attentional biases toward emotions or other stimuli may be partly learned (Guo et al., 2019). Other research has indeed shown that learning effects might modulate the outcomes of a dot-probe task. For instance, previous research has shown that people suffering from alcohol dependency have greater biases toward photographs of alcoholic beverages than control subjects (Townshend & Duka, 2001). Even more strikingly, in non-dependent social drinkers an attentional bias toward alcohol-related stimuli increased after priming with a small (but not large) dose of alcohol (Duka & Townshend, 2004), which shows that even a brief learning episode might already influence attention mechanisms.

When zooming in on conspecific scenes, the results show that humans’ attention was mostly captured by scenes showing sex and yawns. This is partly in line with our earlier study in bonobos where strongest effects were observed in scenes depicting yawns, grooming, and sex (in that order) (Kret et al., 2016). Can we conclude from this that humans are even more attuned to sex than the hyper-sexual bonobo? Unfortunately, we cannot, due to a limitation of this study. That is, the human sexual scenes were standing out from the rest of the images as only that category showed half-naked people. We could not circumvent this problem in the current study as alternative approaches had other drawbacks. For instance, it is difficult to have completely neutral images showing nudes. Even more tricky is to find other emotional scenes showing emotional expressions (e.g. a group of aggressive nudes). That said, future studies should address this confound by using a stimulus category showing nudes in at least relatively neutral poses. The fact that out of all stimulus categories, human adults rated the category sex as most arousing and most positive makes such a follow-up even more appealing.

### Conclusion

Based on participants’ explicit valence and arousal ratings and on their attentional biases toward certain stimulus categories, we can conclude that overall, humans perceive emotional scenes showing people similarly as emotional scenes of bonobos. Especially because this finding was observed in lay people who rarely see bonobos, this effect cannot be explained by learning, but likely reflects a shared evolutionary origin in these expressions themselves.

Some expressions have more communicative potential than others. The smile or the bared teeth display are examples of clear signals, meant for conspecifics to be seen. These expressions can have multiple meanings which can be interpreted correctly within the specific context. We found that children may still need to learn to use these contextual cues when judging a situation as positive or negative.

The sex scenes were rated very positively, especially by male participants. Even though they rated these more positively than women, their attention was captured similarly, by far surpassing all other emotion categories. It is interesting that this sex difference which was observed on an explicit level, disappeared when measured implicitly.

An important consideration when studying emotions comparatively are the differences in species’ physique. Although there are few anatomical differences between humans and bonobos (Diogo, 2018), other differences, such as the fact that bonobos have fur and humans walk bipedally and stand up straight, may make the transmission of certain emotions easier in one species than the other. Bonobos have much larger canines than humans. These canines stand out and could provide a threat signal. Interestingly, humans’ attention was captured more by human yawns than by bonobo yawns, an effect that is more likely to be explained by the highly contagious nature of this stimulus, which is typically enhanced when shown by close others (Palagi et al., 2014). From that point of view, the difference in height between children and adults and their subsequent different viewing angle when reading adults facial expressions, may impact in how these are perceived and learned.

## Supplemental Material

Supplemental Material, sj-pdf-1-evp-10.1177_14747049211032816 - Attentional Bias in Humans Toward Human and Bonobo Expressions of Emotion Click here for additional data file. Supplemental Material, sj-pdf-1-evp-10.1177_14747049211032816 for Attentional Bias in Humans Toward Human and Bonobo Expressions of Emotion by Mariska E. Kret and Evy van Berlo in Evolutionary Psychology
