# Supplementary material for: Attentional Bias in Humans Toward Human and Bonobo Expressions of Emotion
Source: Evol Psychol. 2021 Jul 28;19(3):14747049211032816. doi: 10.1177/14747049211032816 (PMC10358346; doi:10.1177/14747049211032816)

Supplements

Figure 1

Examples of stimuli per emotion category depicting bonobos.

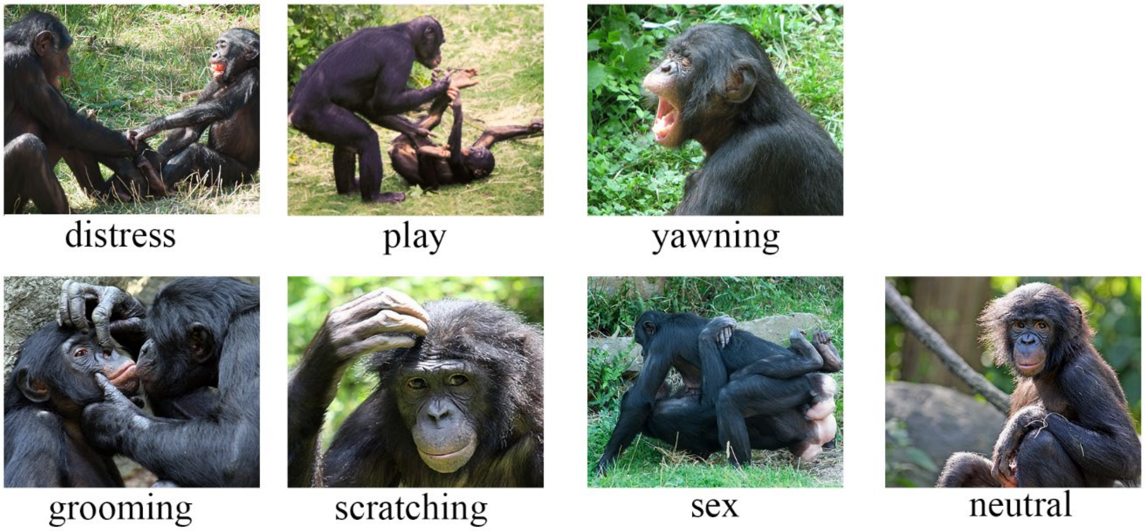

Figure 2

Examples of stimuli per emotion category depicting humans and used in the task with adults and children.

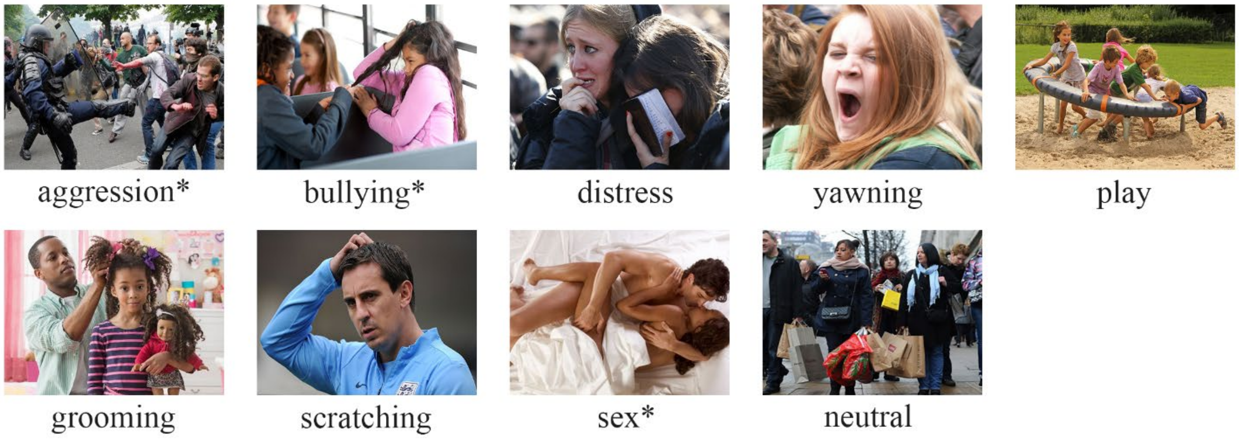

Note. **Aggression was only used in adults, and replaced by bullying in the child version of the task. Sex was removed from the child version.*

Figure 3.

Picture of a child performing one of the dot-probe tasks in the zoo

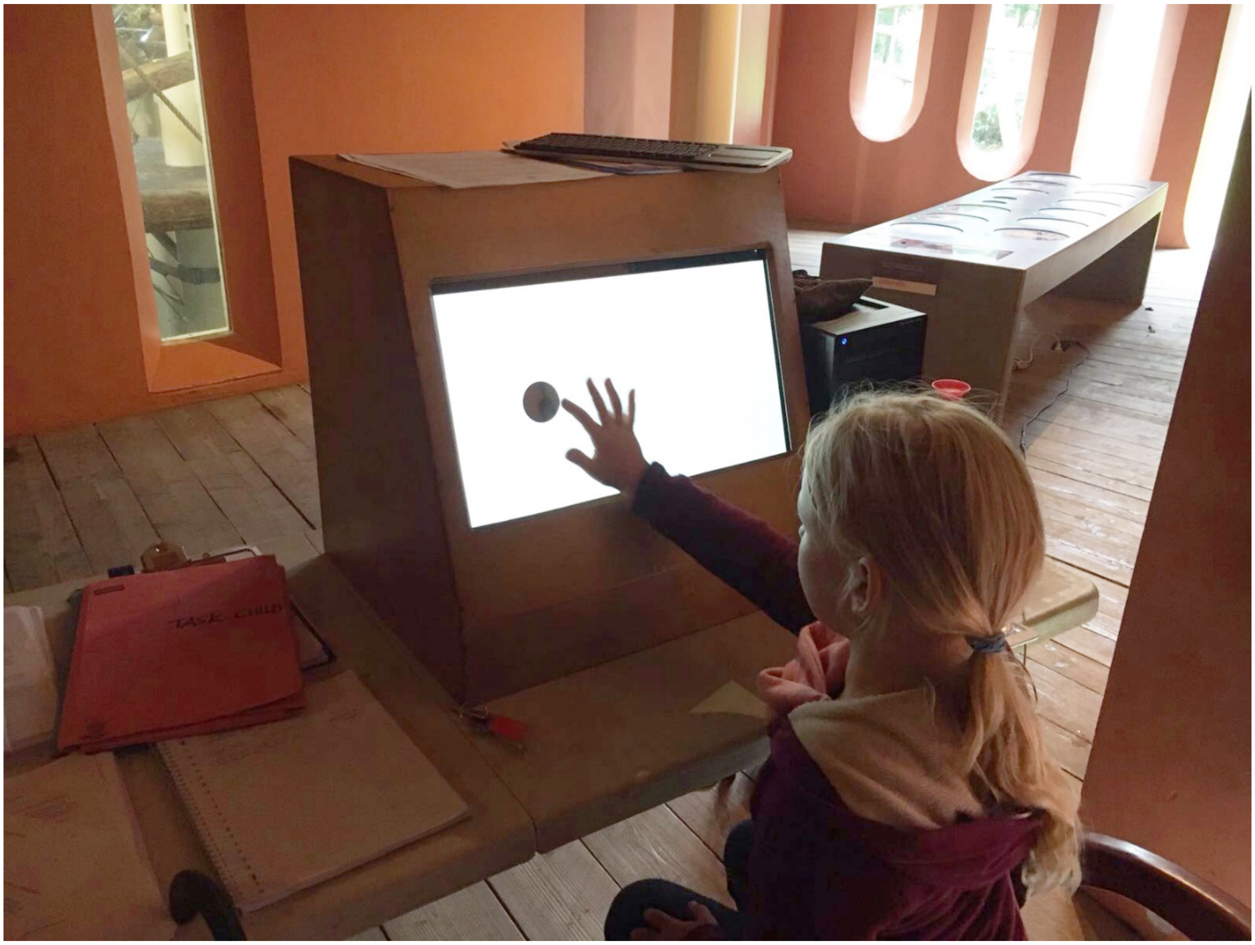

Supplement: Supplemental Material, sj-pdf-1-evp-10.1177_14747049211032816 - Attentional Bias in Humans Toward Human and Bonobo Expressions of Emotion [file sj-pdf-1-evp-10.1177_14747049211032816.pdf]
